# Early Estimates of Monkeypox Incubation Period, Generation Time, and Reproduction Number, Italy, May–June 2022

**DOI:** 10.3201/eid2810.221126

**Published:** 2022-10

**Authors:** Giorgio Guzzetta, Alessia Mammone, Federica Ferraro, Anna Caraglia, Alessia Rapiti, Valentina Marziano, Piero Poletti, Danilo Cereda, Francesco Vairo, Giovanna Mattei, Francesco Maraglino, Giovanni Rezza, Stefano Merler

**Affiliations:** Center for Health Emergencies, Fondazione Bruno Kessler, Trento, Italy (G. Guzzetta, V. Marziano, P. Poletti, S. Merler);; Directorate General of Health Prevention, Ministry of Health, Rome, Italy (A. Mammone, F. Ferraro, A. Caraglia, A. Rapiti, F. Maraglino, G. Rezza);; Directorate General for Health, Lombardy Region, Milano, Italy (D. Cereda);; National Institute of Infectious Diseases Lazzaro Spallanzani, Rome (F. Vairo);; General Directorate for Personal Care, Health and Welfare, Emilia Romagna Region, Bologna, Italy (G. Mattei)

**Keywords:** monkeypox, reproduction number, generation time, incubation period, MPX, MPXV, sexually transmitted infections, Italy, viruses

## Abstract

We analyzed the first 255 PCR-confirmed cases of monkeypox in Italy in 2022. Preliminary estimates indicate mean incubation period of 9.1 (95% CI 6.5–10.9) days, mean generation time of 12.5 (95% CI 7.5–17.3) days, and reproduction number among men who have sex with men of 2.43 (95% CI 1.82–3.26).

After the first reports of autochthonous cases of monkeypox (MPX) in Europe at the beginning of May, the World Health Organization (WHO) and the European Centre for Disease Prevention and Control alerted member states to report suspected and confirmed cases. Apart from early cases reported in the United Kingdom, most cases were identified in men who have sex with men (MSM) (*1*–*4*). We analyzed characteristics of the first 255 PCR-confirmed cases of monkeypox in Italy in 2022.

## The Study

In Italy, suspected MPX cases fitting the criteria of the World Health Organization case-definition (*5*) are reported to the surveillance system of the Ministry of Health. Only those cases testing positive by MPX-specific PCR were considered confirmed. Information on main patient characteristics (age, sex, earliest date of symptom onset, presence of rash and other signs, exposure modality, and travel abroad) were collected.

As of July 8, 2022, a total of 255 PCR-confirmed cases had been reported in Italy (Figure). All except 2 were men, and 190/200 (95%) men who disclosed information reported having sex with men; median age was 37 (range 20–71) years.

**Figure:**
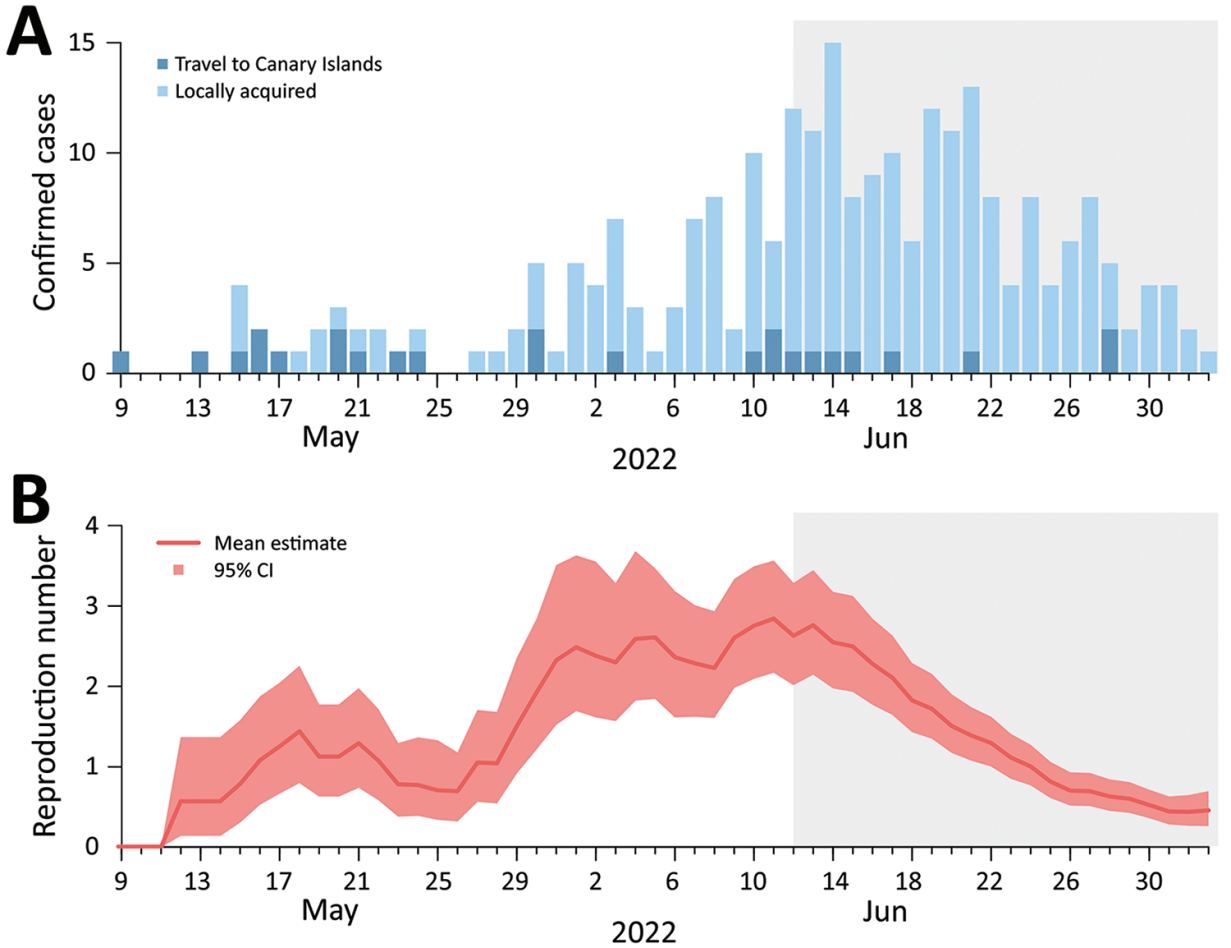
Epidemic curve and reproduction number of monkeypox cases in Italy through July 8, 2022. A) Number of cases by date of symptom onset and history of travel in Canary Islands. For 4 persons, the date of symptom onset was unknown. B) Estimate of the net reproduction number over time from the epidemic curve by date of symptom onset. We assumed that all cases with a history of travel to Canary Islands were imported and that all the others were locally transmitted, and we used a generation time distribution with mean 12.5 days. Gray shading indicates the part of the epidemic curve that is possibly incomplete because of diagnostic and reporting delays.

For 139/184 cases for which information was available, rash was localized at the genital or perianal area. Fever was reported in 151/222 cases for which information was available.

Information about travel was available for 228 case-patients; 86 (37.7%) had traveled abroad, and 25 (29.1%) of those had vacationed in the Canary Islands, suggesting a major amplifying event had occurred (Table). Only 1 case-patient had traveled to West Africa and was symptomatic upon arrival in Italy.

**Table:** Characteristics of 255 confirmed monkeypox cases reported in Italy through July 8, 2022

Characteristic	Value
Sex	
M	253 (99.2)
F	2 (0.8)
Median age, y (range)	37 (20–71)
Clinical symptoms†	
Fever	151/222 (68.0)
Rash	248/251 (98.8)
Genital/perianal rash	139/184 (75.5)
Travels†	
Travel abroad in previous 21 d	86/228 (37.7)
Travel to Canary Islands in previous 21 d	25/142 (17.6)

*Values are no. (%) patients except as indicated. †Denominators indicate the total number of available answers.

We estimated the incubation period for 30 cases with known date of symptom onset and for which epidemiologic investigations enabled the identification of the likely period of exposure (exact date for 15 cases and dates of visit to Canary Islands for 15 cases). We estimated the generation time (time elapsed between date of exposure of a confirmed case and those of secondary cases) by considering 16 infector-infectee pairs identified during contact tracing operations. We assumed the 2 periods were distributed as gamma functions and estimated them using a Bayesian approach similar to that adopted by F. Miura et al. (*6*). We considered likely dates of exposure for each confirmed case within a Markov chain Monte Carlo procedure. For estimating generation time, we assumed no presymptomatic transmission. Therefore, sampling of candidate dates of exposure was repeated if the date of exposure for the infectee was earlier than the date of symptom onset for the infector. We used estimates of the generation time to compute the net reproduction number and used individual anonymized data to estimate the incubation period and generation time (Appendix).

The mean incubation period was estimated to be 9.1 days (95% CI 6.5–10.9 days; 5th and 95th percentiles of the distribution 2–20 days). The mean generation time was estimated to be 12.5 days (95% CI 7.5–17.3 days; 5th and 95th percentiles of the distribution 5–23 days). By assuming a mean generation time of 12.5 days and importation from Canary Islands, we estimate the mean net reproduction number (mean number of cases generated by a single index case) at 2.43 (95% CI 1.82–3.26) during the first week of June (i.e., when the net reproduction number had stabilized so that the growth of the epidemic curve could be approximated as exponential). A similar estimate was obtained under the assumption of exponential growth in the first week of June (Appendix). After June 12, 2022, we estimated a progressive decrease of the reproduction number.

## Conclusions

The first large outbreak of MPX outside Africa is to some extent unique. The analysis of virus genome strongly suggests that the epidemic is caused by the West African clade of the MPX virus (*7*); however, with the exception of 1 case-patient who reported travels to West Africa (*8*), >60% of cases diagnosed in Italy were autochthonous. Retrospective investigations in Portugal and United Kingdom indicated that the first case-patients had symptoms in April 2022. The presence of skin lesions at the point of sexual contact is suggestive of sexual transmission (*9*).

After early reports of this multicountry outbreak, the Ministry of Health of Italy issued recommendations consisting of case notification, protective measures to reduce contacts and possible exposure for healthcare workers, tracing of close contacts with monitoring of symptom onset, and the possibility of implementing quarantine measures at the discretion of local health authorities in particular epidemiologic or environmental contexts (*10*). After the first 4 cases in MSM in Italy who had traveled abroad (*4*), cases were increasingly notified (*8*), mostly in the Lombardy and Lazio regions, where Milan and Rome are located; 11 of 21 regional health authorities reported cases. Almost all cases were among MSM. Travel abroad occurred in a substantial fraction of cases (38.9%) identified in Italy, and direct or sexual contact is still likely to be the main transmission mode. Whether the infection was transmitted through direct contact with skin lesions or body fluids remains undefined. The link to different geographic areas (Europe and West Africa) underlines the possibility of multiple independent introductions of the virus, suggesting widespread infection in West Africa before the COVID-19 pandemic (*3*,*11*).

Using a limited number of MPX cases, we provided estimates of the mean incubation period (≈9 days; n = 15 persons with known date of exposure and 15 persons with known travel dates in Canary Islands) and of the mean generation time (≈12 days; n = 16 infector-infectee pairs). Based on the estimated mean generation time, we found that the reproduction number for this outbreak is ≈2.4, although with a broad uncertainty (95% CI 1.82–3.26) because of the limited number of locally acquired confirmed cases. We found small variations in the estimated reproduction number (mean values ranging from 2.08 to 2.70) when considering different distributions of the generation time (mean 7.5 or 17.3 days) and when exploring alternative assumptions on the importation of cases (Appendix). Our estimates of the reproduction number refer to the community of MSM in which MPX is spreading and not to the general population. The extent to which the decrease of the reproduction number estimated after June 12, 2022, is a result of reduced transmission (e.g., led by increasing awareness about the risk of infection) or from the analysis of incomplete data because of diagnostic and reporting delays is unclear. However, considering that most cases seem to have been transmitted by sexual contact, the reproduction number is likely below threshold in the general population. Besides the limited number of cases, our estimates might be biased by several factors: the assumption that case-patients returning from Canary Islands acquired the infection there, possible recall bias for the dates of exposure, and selection bias in the reconstructed infector-infectee pairs (e.g., a recent sexual partner might be more likely to be identified). Finally, the observed reproduction number might have been inflated by the potential occurrence of superspreading events.

Maintaining a high level of public attention and providing nonstigmatizing information to at-risk population groups are key to contain the spread of MPX virus, in addition to considering the seasonal intensity of aggregation events and recreational activities. Our estimates provide useful indications to assist with outbreak surveillance and containment. The distribution of the incubation period identifies the period over which symptoms should be monitored among identified contacts, and the generation time provides insight on the recommended duration of isolation for confirmed cases and the timeframe for contact tracing. The generation time is also necessary for computing the net reproduction number, which is critical to monitoring the spread of disease over time.

Appendix Additional information about early estimates of monkeypox incubation period, generation time, and reproduction number, Italy, May–June 2022
