# Supplementary material for: Early Estimates of Monkeypox Incubation Period, Generation Time, and Reproduction Number, Italy, May–June 2022
Source: Emerg Infect Dis. 2022 Oct;28(10):2078–81. doi: 10.3201/eid2810.221126 (PMC9514338; doi:10.3201/eid2810.221126)
Supplement: Appendix — Additional information about early estimates of monkeypox incubation period, generation time, and reproduction number, Italy, May–June 2022 [file 22-1126-Techapp-s1.pdf]

Article DOI: https://doi.org/10.3201/eid2810.221126

Early Estimates of Monkeypox Incubation Period, Generation Time, and Reproduction Number, Italy, May–June 2022

Appendix

Incubation Period

To compute the incubation period, we considered all monkeypox cases confirmed through July 8, 2022, with a known date of symptom onset and for which ≥1 of the following information was reported:

• the latest day of exposure, as ascertained by contact tracing investigation (this information is available for 15 cases);

• a history of travel to Canary Islands, together with the dates of sojourn (this information is available for 15 cases).

As a baseline, we thus considered a total of 30 confirmed cases with prior information on the possible date of exposure (Appendix Table 1).

We assumed that the incubation period is distributed as a gamma function and we estimated the shape (k) and scale parameters (θ) following a Bayesian approach similar to the one adopted in Miura et al. (*1*), based on a Monte Carlo Markov Chain (MCMC) procedure and Metropolis-Hastings sampling. At each MCMC iteration:

• we sampled 1 date of infection for each confirmed case, considering the information available for each subject. For cases with a known latest day of exposure, the date of infection was fixed to this value; for cases with a travel history to Canary Islands, the sampled date of infection was constrained to be between the date of departure to and return from Canary Islands.

• for each confirmed case, we computed the incubation periods as the difference between the date of symptom onset and the sampled date of infections.

• we sampled 1 value for the shape (k) and 1 value for the scale parameter (θ) of the gamma function.

• we compute the gamma likelihood of observing the computed incubation periods given parameters $\{k, \theta\}$

The resulting estimates of the parameters are reported in Appendix Table 2. The cumulative density function estimated for the incubation period is shown in Appendix Figure 1 along with the cumulative distribution of incubation periods associated to the parameter set with the maximum likelihood.

As sensitivity analyses, we estimated the incubation period by considering separately the 15 cases with a known latest date of exposure (sensitivity A) and the 15 cases with a history of travel to Canary Islands (sensitivity B).

Generation Time

The generation time is defined as the difference between the date of infection of a confirmed case and those of secondary cases. To estimate the generation time, we followed a MCMC procedure similar to the one used for the incubation period, by assuming a gamma distributed generation time. In the data, there were 16 identified infector-infectee pairs with known dates of symptom onset (Appendix Table 3). For 2 cases, an exact date of last exposure was identified during epidemiologic investigations and therefore assumed as infection date. For 1 case-patient who reported traveling to Canary Islands but had no other known exposure, we constrained the sampled infection date to be within the dates of departure to and return from Canary Islands. For other cases, the dates of infection were randomly sampled assuming that presymptomatic transmission is not possible (i.e., the date of infection for the secondary cases should always be greater or equal to the date of symptom onset of the infector). Dates of infection were sampled using as a uniform prior between values lying within the 95% CI of the incubation period estimated in the baseline analysis.

The obtained distribution of the generation time is reported in Appendix Figure 2, and the values are summarized in Appendix Table 4.

Reproduction number R_t

We computed the net reproduction number R_t for the monkeypox outbreak using the epidemic curve of cases by date of symptom onset and the estimated distribution of the generation time, by applying a standard statistical method based on the renewal equation. The posterior distribution of R_t can be computed by applying a MCMC algorithm to the following likelihood function:

$$\mathcal{L} = \prod_{t=1}^{T} P\left(C(t) - I(t);\ R_t \sum_{s=1}^{T} \varphi(s) C(t-s) \right)$$

Where:

• $P(k; \lambda)$ is the probability mass function of a Poisson distribution (i.e., the probability of observing k events if these events occur with rate λ).

• $C(t)$ is the total daily number of new cases having symptom onset at time t;

• $I(t)$ is the total daily number of new cases that are not locally transmitted;

• R_t is the net reproduction number at time t to be estimated;

• $\varphi(s)$ is the probability distribution density of the generation time discretized by day, evaluated at days s.

We considered 3 different assumptions for imported cases:

1) we considered only the case with earliest symptom onset as an imported case. This is a rather unrealistic hypothesis that can give an upper bound to the transmissibility of monkeypox;

2) we considered as imported cases all cases with a history of travel to Canary Islands; this assumption was considered as a baseline for the results in the main text;

3) we considered as imported cases all cases with a history of travel abroad; this is also an unrealistic hypothesis that can be considered as a lower bound to the transmissibility of monkeypox.

Cases for which there are no information for travel abroad are always assumed to be locally transmitted.

In Appendix Figure 3, we report estimates of the reproduction numbers for the 3 hypotheses (rows) as obtained by considering the mean and the lower and upper limits of the 95% CI of the generation time, namely 12.5 days, 7.5 days, and 17.3 days (columns). Appendix Table 5 reports the values of the average reproduction number during the first week of June (i.e., the last week for which the epidemic curve can be reasonably assumed to not suffer from diagnostic and reporting delays).

As a sensitivity analysis, we estimated the reproduction number under the assumption of exponential growth in the first week of June, computed from the formula above by forcing Rt to be constant over the first week of June. Appendix Table 6 shows the estimated value (mean and 95% CI) for all combinations of assumptions on imported cases and mean generation time. The values are in line with those of the main analysis, reported in Appendix Table 5.

References

1. Miura F, van Ewijk CE, Backer JA, Xiridou M, Franz E, Op de Coul E, et al. Estimated incubation period for monkeypox cases confirmed in the Netherlands, May 2022. Euro Surveill. 2022;27:2200448. PubMed https://doi.org/10.2807/1560-7917.ES.2022.27.24.2200448

Appendix Table 1. Information on possible dates of monkeypox exposure for 30 cases*

ID	Type of information	Date of symptom onset	Date of earliest exposure	Date of latest exposure
i1	Travel to Canary Islands	8	6	8
i2	Travel to Canary Islands	12	9	12
i3	Travel to Canary Islands	14	2	14
i4	Travel to Canary Islands	15	8	15
i5	Travel to Canary Islands	15	11	14
i6	Travel to Canary Islands	19	11	16
i7	Travel to Canary Islands	20	7	14
i8	Travel to Canary Islands	22	9	14
i9	Known exposure date	23	18	18
i10	Travel to Canary Islands	23	11	17
i11	Known exposure date	28	25	25
i12	Known exposure date	29	27	27
i13	Travel to Canary Islands	29	7	17
i14	Travel to Canary Islands	29	7	14
i15	Travel to Canary Islands	33	9	14
i16	Known exposure date	37	31	31
i17	Known exposure date	38	31	31
i18	Known exposure date	40	21	21
i19	Known exposure date	42	35	35
i20	Travel to Canary Islands	47	32	46
i21	Known exposure date	48	42	42
i22	Known exposure date	49	40	40
i23	Known exposure date	49	40	40
i24	Known exposure date	50	46	46
i25	Known exposure date	51	40	40
i26	Known exposure date	51	40	40
i27	Known exposure date	52	41	41
i28	Known exposure date	54	44	44
i29	Travel to Canary Islands	58	49	56
i30	Travel to Canary Islands	58	44	51

*Dates are expressed in days from a reference date that is not made explicit to preserve anonymity.

Appendix Table 2. Parameters of the incubation period distributions estimated using different methods

Method	Baseline (n = 30)	Sensitivity A (n = 15)	Sensitivity B (n = 15)
Shape (95% CI)	2.42 (1.26–3.62)	3.55 (1.57–6.38)	1.43 (0.62–2.96)
Scale (95% CI)	3.75 (2.17–7.13)	2.67 (1.21–5.73)	7.47 (2.97–16.84)
Mean, d (95% CI)	9.1 (6.5–10.9)	9.5 (6.3–11.2)	10.7 (5.5–14.5)
5th and 95th percentiles of the distribution, d	2–20	3–19	1–28

Appendix Table 3. Information on infector-infectee pairs*

Infector				Infected			
ID	Symptom onset	Earliest exposure	Latest exposure	ID	Symptom onset	Earliest exposure	Latest exposure
g1	9	1	6	g17	18	11	11
g2	9	NA	NA	g18	30	15	15
g3	23	NA	NA	g19	25	NA	NA
g4	27	NA	NA	g20	41	NA	NA
g5	27	NA	NA	g21	39	NA	NA
g6	29	NA	NA	g22	29	NA	NA
g7	31	NA	NA	g23	34	NA	NA
g8	32	NA	NA	g24	43	NA	NA
g9	33	NA	NA	g25	40	NA	NA
g10	33	NA	NA	g26	40	NA	NA
g11	34	NA	NA	g27	39	NA	NA
g12	34	NA	NA	g28	37	NA	NA
g13	35	NA	NA	g29	51	NA	NA
g14	36	NA	NA	g30	46	NA	NA
g15	37	NA	NA	g31	41	NA	NA
g16	44	NA	NA	g32	51	NA	NA

*Dates are expressed in days from a reference date that is not made explicit to preserve anonymity. NA, not applicable.

Appendix Table 4. Parameters of the generation time distributions.

Generation time (n = 16)	Value
Shape (95% CI)	4.85 (3.07−7.20)
Scale (95% CI)	2.57 (1.34−4.07)
Mean (95% CI), d	12.5 (7.5–17.3)
5th and 95th percentiles of the distribution, d	5–23

Appendix Table 5. Estimates of the net reproduction number (mean and 95% CI) in the week of June 1–7 as obtained by considering the mean (first column), lower (central column), and upper (right column) limits of the 95% CI of the generation time

	Mean generation time		
Assumed imported cases	12.5 d (shape: 4.85, scale: 2.57)	7.5 d (shape: 6.86, scale: 1.10)	17.3 d (shape: 5.67, scale: 3.05)
Single importation	2.51 (1.90−3.18)	2.34 (1.77−3.00)	2.70 (2.04−3.37)
Importations from Canary Islands, baseline	2.43 (1.82–3.26)	2.24 (1.71–2.99)	2.63 (1.96–3.41)
Importations from abroad	2.23 (1.61–2.92)	2.08 (1.50–2.80)	2.41 (1.77–3.16)

Appendix Table 6. Estimates of the reproduction number (mean and 95%CI) under the assumption of exponential growth in the week of June 1–7, as obtained by considering the mean (first column), lower (central column), and upper (right column) limits of the 95% CI of the generation time

	Mean generation time		
Assumed imported cases	12.5 d (shape: 4.85, scale: 2.57)	7.5 d (shape: 6.86, scale: 1.10)	17.3 d (shape: 5.67, scale: 3.05)
Single importation	2.71 (1.67–3.66)	2.31 (1.42–3.15)	3.06 (1.88–4.13)
Importation from Canary Islands, baseline	2.50 (1.71–3.40)	2.14 (1.46–2.95)	2.82 (1.92–3.85)
Importation from travel abroad	2.40 (1.61–3.45)	1.96 (1.33–2.85)	2.75 (1.81–3.86)

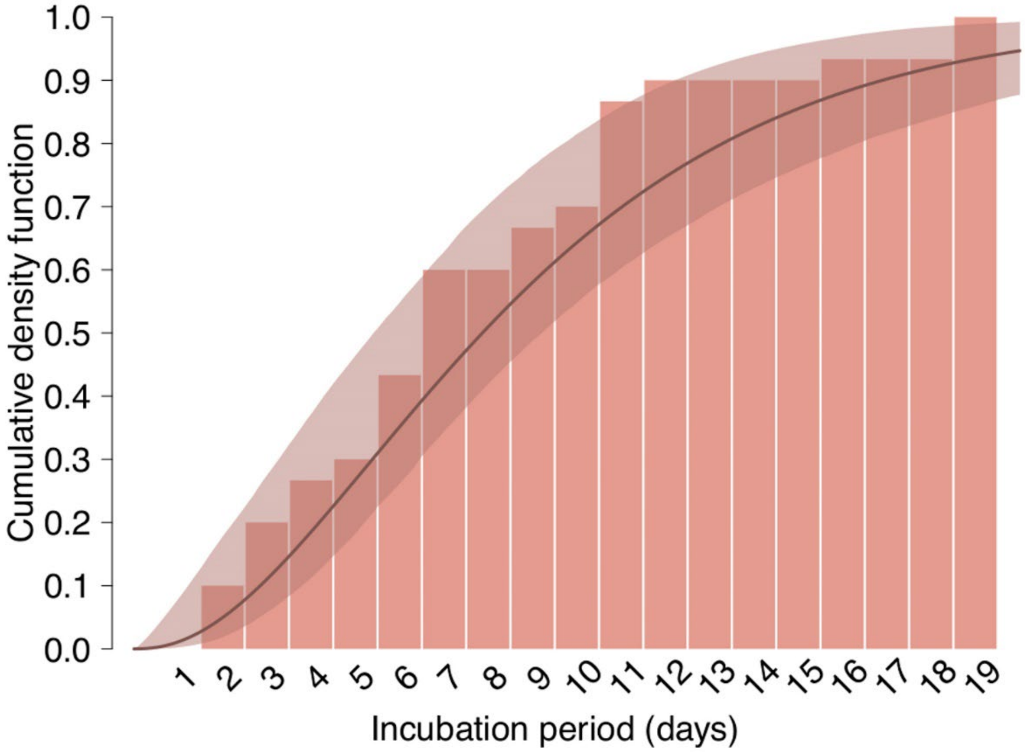

Appendix Figure 1. Cumulative density function of the incubation period of monkeypox as estimated from 30 cases confirmed in Italy during May–June 2022 (mean, solid line; 95% CI, shaded areas). Bars represent the cumulative distribution of the incubation periods as obtained for these 30 confirmed cases, according to the maximum likelihood parameter set.

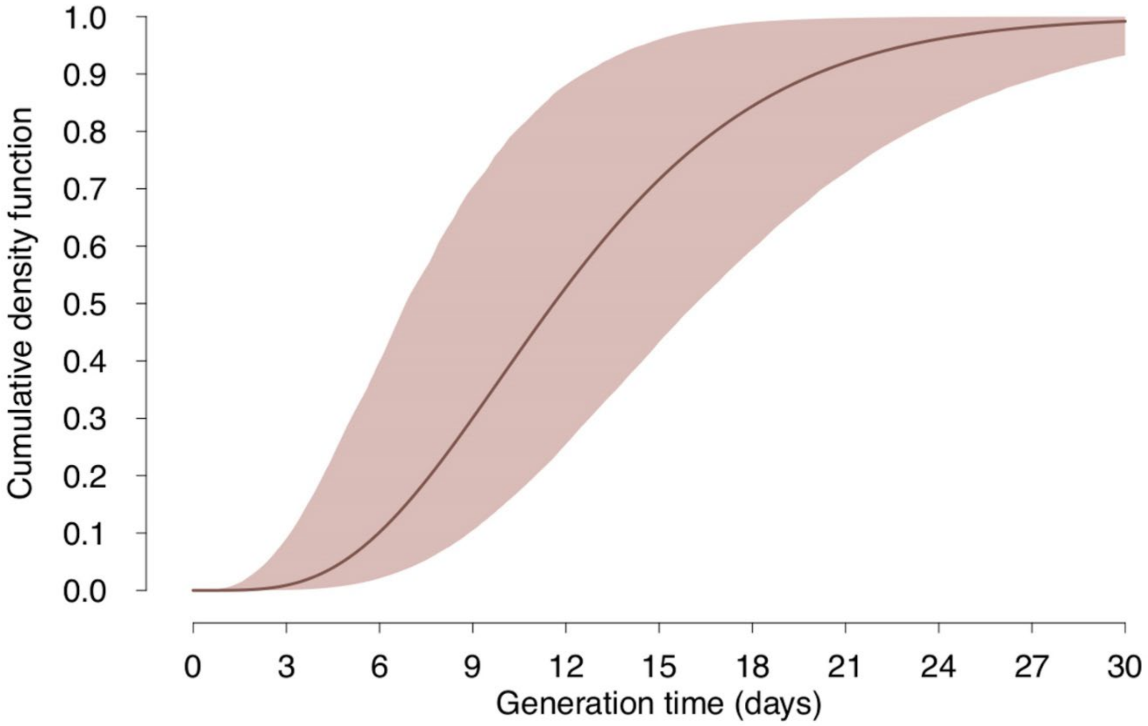

Appendix Figure 2. Cumulative density function of the generation time of monkeypox as estimated from 16 infector-infectee pairs identified during contact tracing operations conducted in Italy during May–June 2022 (mean, solid line; 95% CI, shaded areas).

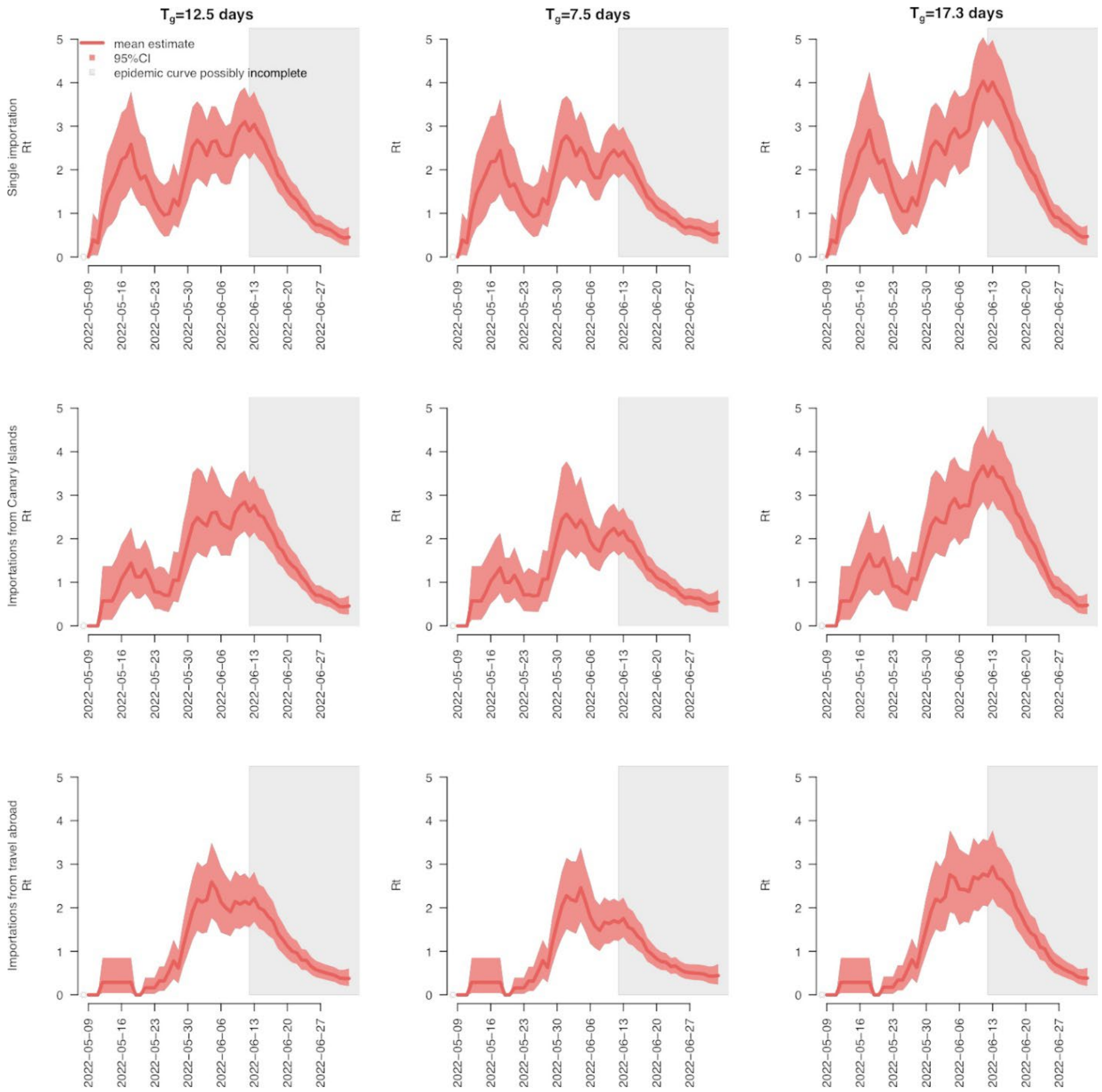

Appendix Figure 3. Net reproduction number (mean and 95% CI) under the assumption that only the first case was imported (top row), that all cases with a history of travel to Canary Islands were imported (middle row), or that all cases with a history of travel abroad were imported (bottom row). Different distributions of the generation time were used: the first distribution (left column) has mean 12.5 days (corresponding to mean estimates reported in Appendix Table 4); the second distribution (center column) has mean 7.5 days (corresponding to the lower bound of the 95% CI provided in Appendix Table 4); the third distribution (right column) has mean 17.3 days (corresponding to the upper bound of the 95% CI provided in Appendix Table 4). The baseline results for the main text are those assuming importation from Canary Islands and a mean generation time of 12.5 days (middle row, left column).
